# Design and validation of a real-time cassava planter seed quality monitoring system based on optical fiber sensors and rotary encoders

**DOI:** 10.3389/fpls.2024.1481909

**Published:** 2024-12-03

**Authors:** Bin Yan, Zhende Cui, Ganran Deng, Guojie Li, Shuang Zheng, Fengguang He, Ling Li, Pinlan Chen, Xilin Wang, Sili Zhou, Ye Dai, Shuangmei Qin, Zehua Liu

**Affiliations:** ^1^ Agricultural Machinery Research Institute, Chinese Academy of Tropical Agricultural Sciences, Zhanjiang, Guangdong, China; ^2^ Key Laboratory of Tropical Agricultural Machinery, Ministry of Agriculture and Rural Affairs, Zhanjiang, Guangdong, China

**Keywords:** cassava combine planter, cassava seed stalk, seeding quality monitoring, fault monitoring, sensor

## Abstract

Targeting the issues of seed leakage and cutting segment adhesion due to poor seed feeding and cutting in real-time seed-cutting cassava planters, this study developed a seeding quality monitoring system. Based on the structure and working principle of the seed cutting and discharging device, the installation methods of the matrix fiber optic sensor and rotary encoder were determined. By combining the operational characteristics of the planter’s ground wheel drive with seed cutting and seed dropping, a monitoring model correlating the sowing parameters with seed dropping time was established; a monitoring window was created by extracting and processing the rotary encoder pulse signal, and the number of seeds sown after each opposing cutter’s operation was calculated based on the pulse width information within the monitoring window. The monitoring system’s statistics were compared and analyzed with the manual statistics, and the bench test showed that the monitoring system designed in this study offers high accuracy. When the simulated rotational speed of the opposing cutter ranges from 10 to 30 rpm, the average monitoring error between the monitored and actual seeding quantities for the left and right rows is less than 1.4%. The monitoring system can promptly and accurately activate sound and light alarms for faults, achieving a 100% success rate in alarms and an average fault response time of less than 0.4 seconds. Field tests demonstrate that the average error in seeding volume is 0.91%, and the monitoring system can timely alert to faults occurring in the planter. The system fulfills the requirements for real-time monitoring of cassava seeding volume at various operating speeds in field conditions, and can serve as a reference for monitoring operational parameters in subsequent cassava combine harvesters.

## 1 Introduction

Cassava is a crucial crop extensively cultivated in tropical and subtropical regions, playing a vital role in global food security and economic development (He et al., 2023; Chenna et al., 2024). Seeding quality, as a fundamental aspect of cassava production, directly influences both crop yield and health (Cay et al., 2018; Albarenque et al., 2023). Due to its benefits in enhancing sowing accuracy, crop yield, and farmland management (Rossi et al., 2023), precision sowing technology has become integral to modern agricultural practices (Tang et al., 2022; Ling et al., 2024; Jin et al., 2024a). With ongoing advancements in agricultural mechanization, real-time cut-seed sowing technology is increasingly being employed in cassava planting to address various challenges associated with mechanized sowing (Lungkapin et al., 2009; Chen et al., 2023). However, when a cassava planting machine operates in a fully closed state, the operator cannot directly monitor seeding quality (Bai et al., 2022), leading to issues such as seed leakage and cut-section sticking (Xing et al., 2020), which reduce cassava yield (Wang et al., 2020). Consequently, developing an efficient seeding quality monitoring system is essential.

Failures affecting planter seeding can arise from various factors, including the planter, seed stalk, and operator. The length of individual seed poles is non-standardized, making it difficult to regulate the cutting length accurately at the end of the cut; seed stalks can become bent due to typhoons, preventing their timely and efficient transfer to the cutter position; the operator failed to fill the seed rods to the seed release point in a timely manner during the planting process while the planter continued to move forward; all these factors may result in planting faults. Prolonged and high-intensity operation of the planter can lead to a shift in the relative position of the opposing cutter or a reduction in sharpness, resulting in incomplete cuts that cause adhesion. Real-time monitoring of seeding passes and failures, along with timely feedback to operators and farmers, plays a crucial role in ensuring high cassava yields and accurately predicting crop yields (Karimi et al., 2019).

The performance of the seeding quality monitoring system reflects the seeding effectiveness of planters in real time. To achieve effective seeding quality monitoring, the design of the monitoring system and the integration of advanced sensor technologies are essential. Recent advancements in sensor technology have significantly improved the potential for enhancing seeding quality monitoring (Zhao et al., 2023; Jin et al., 2024). Optical sensors, known for their low cost and high accuracy, have become prevalent in precision agriculture (Xie et al., 2021a). For instance, Wang et al. developed a sowing quality monitoring system for small-sized seeds using an opposed matrix fiber optic sensor, which achieved a counting relative error of no more than 4.6% and was capable of issuing alerts when a seed hole was blocked (Wang et al., 2022). Tang et al. employed diffuse reflective optoelectronic sensors and rectangular fiber optic sensors to monitor seed counts during the maize sowing process and observed a decline in monitoring accuracy as the rotational speed of the seed metering disk increased (Tang et al., 2022). Additionally, other researchers have explored various monitoring techniques, including image recognition, piezoelectric, and capacitive methods, to enhance the seeding process (Wang et al., 2019; Bai et al., 2021; Zhang et al., 2022; Xu et al., 2023). However, image recognition methods encounter challenges related to handling large volumes of data, complex structures, and higher costs during application. Meanwhile, the monitoring accuracy of piezoelectric and capacitive sensing technologies is highly susceptible to interference from environmental factors such as vibration, temperature, and humidity, leading to significant fluctuations in accuracy, these methods face practical limitations in field applications (Jiang et al., 2021; Chen et al., 2019).

Examining the current status of existing research on seeding monitoring systems, it is evident that most research focuses on the monitoring of small-sized seeds (Basso et al., 2016; Zhao et al., 2018; Xie et al., 2021b), and the quality of sowing is predominantly assessed by estimating the number of seeds, rather than their exact count. Cassava is an asexually propagated crop (Sheat et al., 2024)with large seed fragments that result in poor mobility, and the uneven terrain in cassava-growing areas complicates the accurate control of the planter’s forward speed (Chen et al., 2022; Xie et al., 2023), making it difficult to evaluate the quality of monitoring using the existing system. Furthermore, cassava seeders are prone to two types of operational faults: missed seeding and cut-seed sticking. Existing seeding quality monitoring systems typically detect only two types of faults: missed sowing and re-sowing (Xie et al., 2022), and lack a real-time model and system for monitoring cut-seed sticking. This makes it challenging to effectively meet the real-time monitoring needs of cassava planting.

To address these issues, this study focuses on the accurate monitoring of sowing parameters for cassava planters, and designs a real-time cut-section sowing quality monitoring system incorporating a rotary encoder and a matrix fiber optic sensor. By extracting and processing pulse signals to obtain temporal characteristic parameters within the monitoring window, seeding parameters are accurately calculated, and the system’s effectiveness and reliability were evaluated through bench and field tests. The real-time seed cut seeding quality monitoring scheme proposed in this study offers valuable insights for the development and optimization of seeding quality monitoring systems.

## 2 Materials and methods

### 2.1 Research carrier

**Figure 1:**
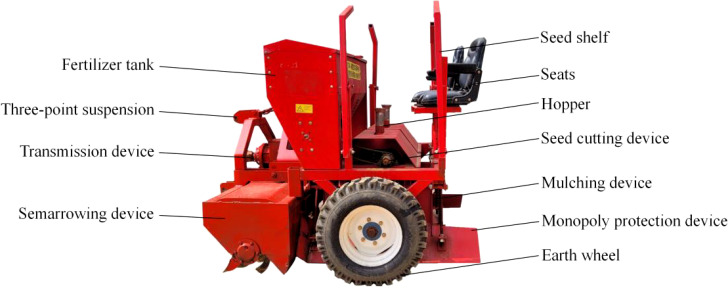
Schematic diagram of cassava combine planter.

The entire machine structure, as depicted in **Figure 1**, is designed by the research team as the research carrier for the monitoring system: the starter cassava joint planter. The planter primarily consists of a seed cutting device, a ridging device, a ridging protection device, a mulching device, a fertilizer discharge device, a ground wheel driving device, a transmission device, and a seed frame. The machine employs a wide and narrow double-row planting mode, with row spacing of 50 to 70 cm, a width of 160 to 180 cm, plant spacing of 60 to 80 cm, row height of 25 to 30 cm, and sowing depth of 5 to 10 cm. It can complete a one-time cassava sowing operation for 1 or 2 rows. The planting machine performs real-time seed cutting and sowing operations through a three-point suspension device under the tractor’s traction. During operation, the cassava seed stems are manually placed into the seeding area. The ridging device creates rows under the drive of the transmission system, the furrow opener forms grooves in the rows, and simultaneously, the ground wheel driving device rotates the seed cutting device to cut the cassava seed stems in real time. These cut stems then fall into the grooves, completing sowing through the seed guide tube. Finally, the mulching device, protected by the ridge guarding device, covers the soil in the grooves to complete mulching. This planting machine can simultaneously perform the functions of ridging, furrowing, fertilizing, and planting, significantly improving planting efficiency to 4-6 mu per hour, and meeting the mechanized planting needs of cassava.

### 2.2 Design of seeding quality monitoring system

#### 2.2.1 System fundamentals

**Figure 2:**
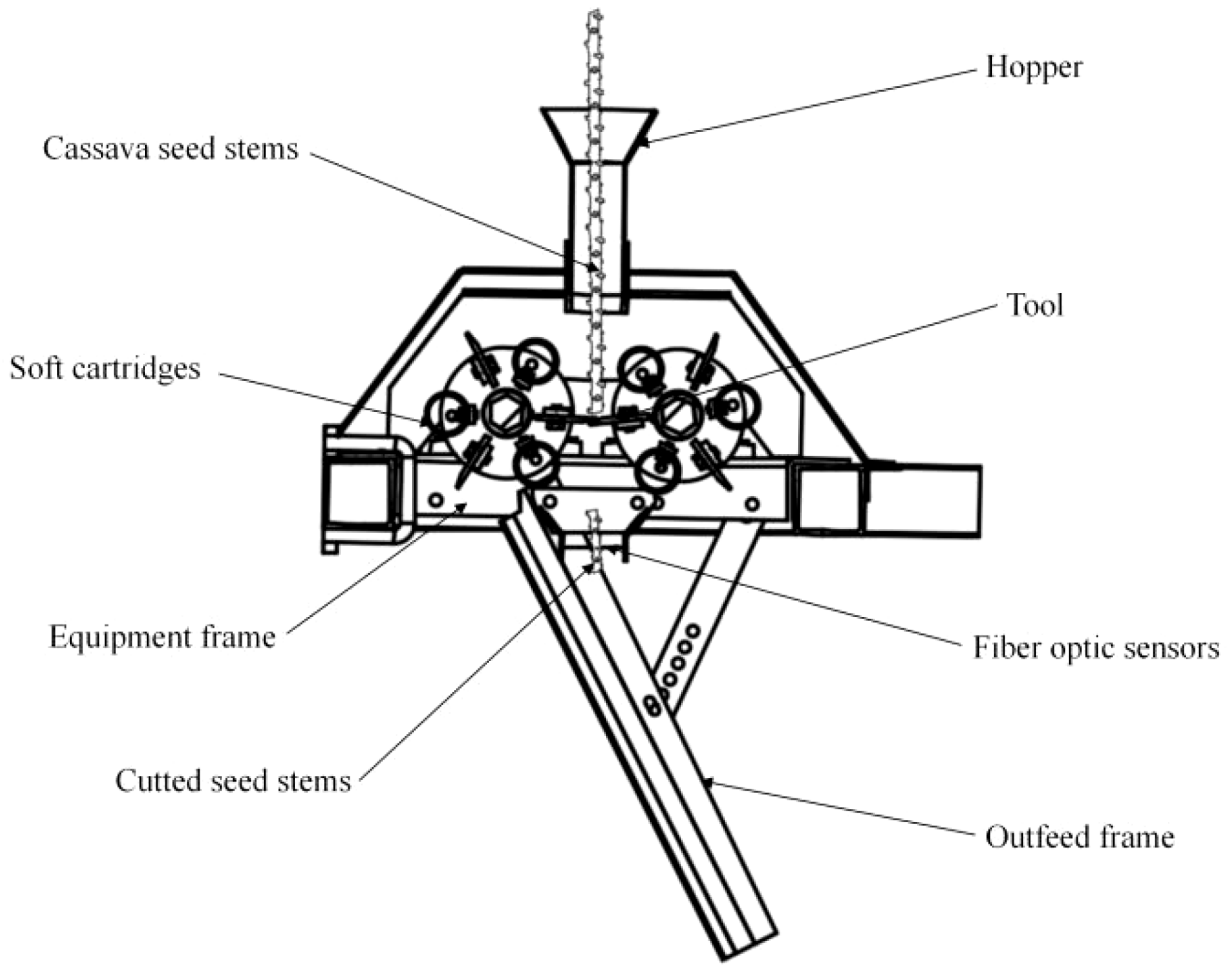
Simplified sketch of the arrangement of the seed cutting device and the seed discharge device.

The working process of the real-time seed cutting and discharging unit can be divided into four stages: seed release, transmission, seed cutting, and seed discharging, as shown in **Figure 2**. During operation, manually place the seed stems into the seed release hopper. The seed stems are then transported to the seed cutting area via clamping and transmission by the soft cartridges. The opposite cutter, driven by the ground wheel drive device, cuts off the cassava seed stems, and the cassava seeds are discharged by gravity. The real-time seed discharge process involves a simple hopper structure positioned close to the cutter. The seeds, after being cut, complete the discharge process through the hopper, undergoing free-fall movement, which is more stable. Therefore, it is preferable to install a seeding monitoring sensor in the hopper. Fiber optic sensors exhibit high sensitivity, strong anti-interference capabilities, and robust environmental adaptability, enabling stable performance in complex operating environments. This makes them highly effective for real-time, high-speed seed cutting and precise monitoring of cassava seeds. To ensure accurate monitoring of the rotational angle of the opposite cutter, the encoder is directly mounted on the rotary axis of the opposite cutter via a fixed bracket.

**Figure 3:**
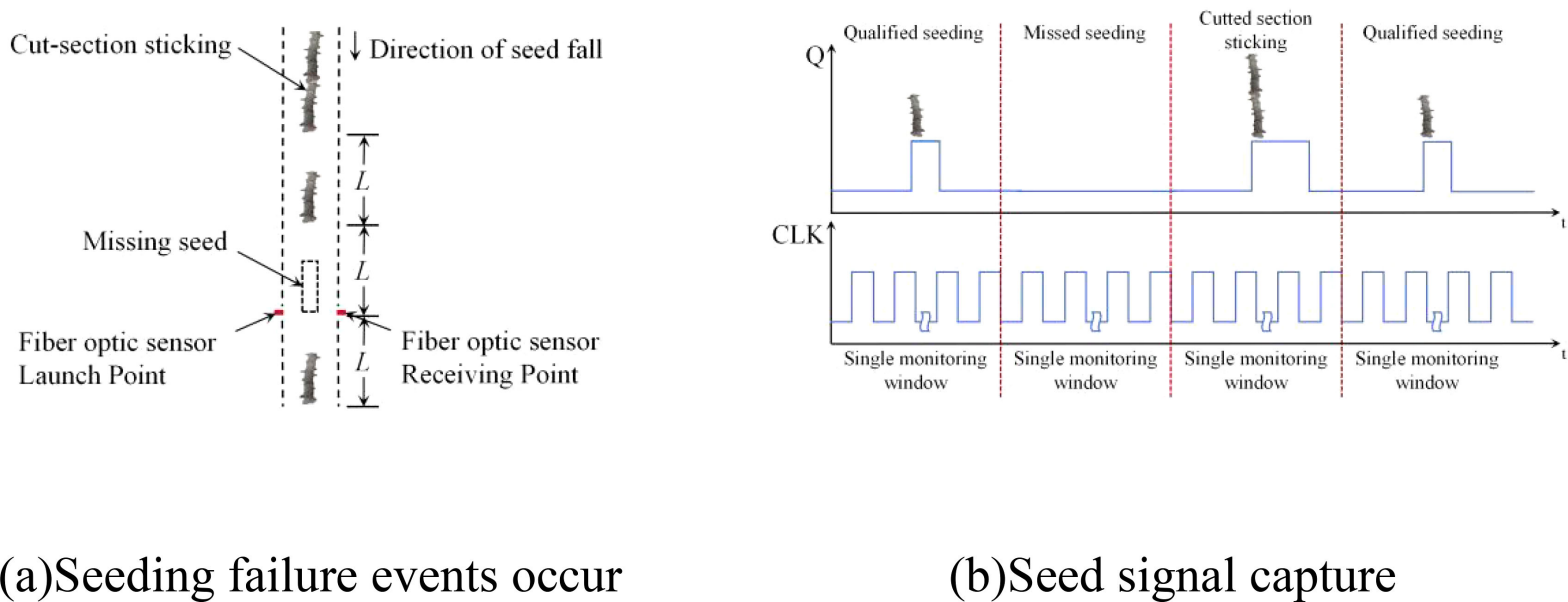
Seeding quality monitoring schematic. **(A)** Seeding faults. **(B)** Window monitoring principle.

During seed rowing, cassava seeds flow through the photoelectric sensor monitoring area, causing variations in the intensity of the infrared beam, which in turn leads to changes in the voltage of the photoresistor at the receiving end, as illustrated in **Figure 3A**. The microcontroller employs the input capture interrupt to collect the pulse signals generated by cassava seeds flowing through the matrix fiber optic sensor group, thereby obtaining the seed rowing time sequence. The angle-splitting node is determined by monitoring the number of pulses from the rotary encoder. The sowing number monitoring window is established by two neighboring angle-splitting nodes, with each monitoring window corresponding to one sowing number, as illustrated in **Figure 3B**. The pulse width within the single monitoring window is analyzed to determine the seeding number. If there is a sowing leakage or a fault in the cutting section, the pulse width within the monitoring time window deviates from the theoretical threshold range, triggering the alarm circuit to activate both sound and light alarms (Wang et al., 2020).

#### 2.2.2 Hardware system design

**Figure 4:**
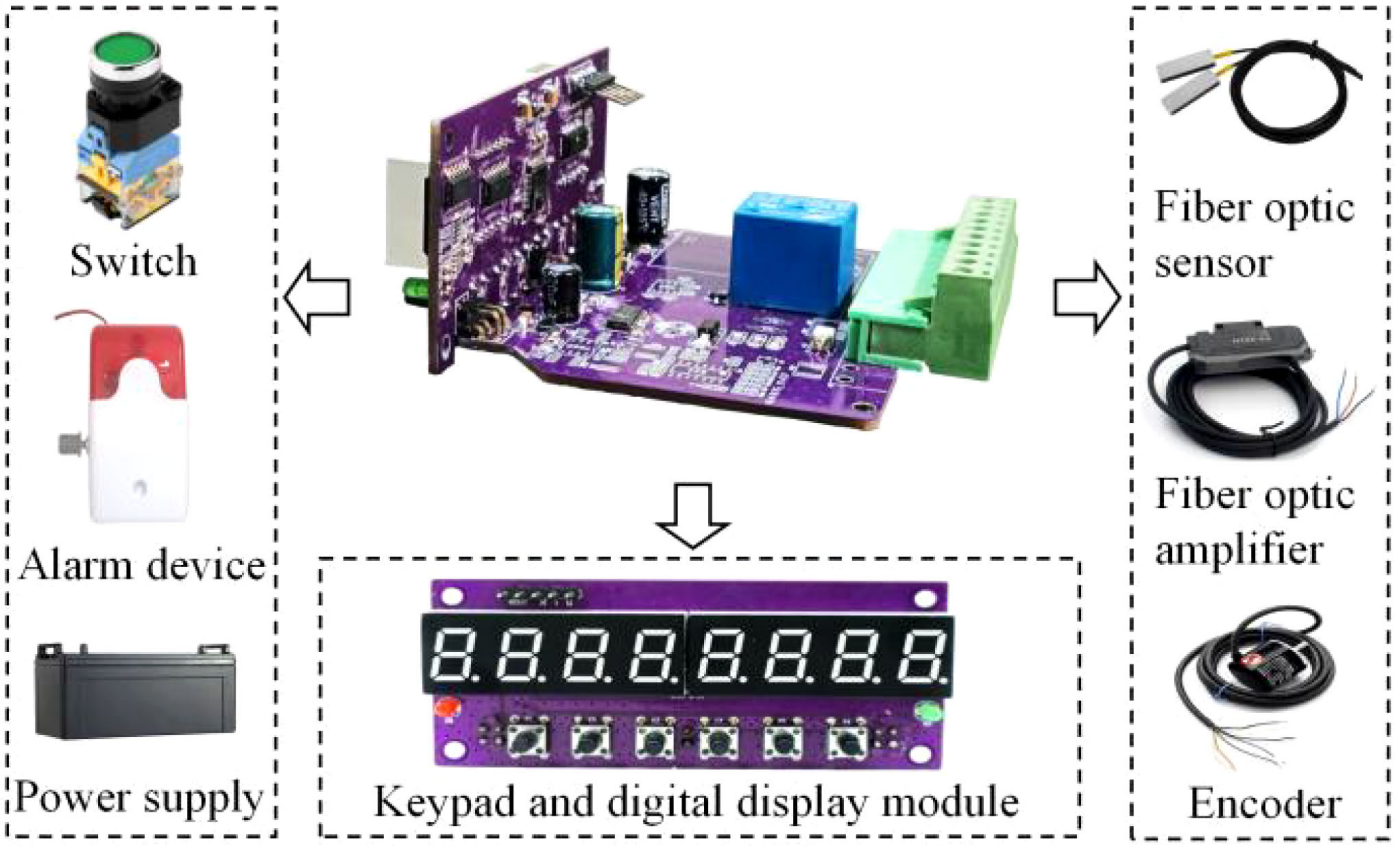
Schematic diagram of the hardware of the seeding quality monitoring system.

The hardware system primarily consists of a microcontroller, an opposed matrix fiber optic sensor, a fiber optic amplifier, an incremental rotary encoder, an acousto-optic alarm, a keypad, and a digital display module, as illustrated in **Figure 4**. The microcontroller utilizes the ARM Cortex-M4-based N32G430C8L7 microprocessor as the primary control chip. It operates at a main frequency of 128 MHz and is equipped with 64 KB flash memory, 16 KB SRAM, 8 timers, and communication interfaces including 4 U(S)ART, 2 SPI, 2 IIC, and 1 CAN. This configuration adequately supports the high-speed seeding flow monitoring required for cassava in this study. The matrix fiber optic sensor, FT-70S, equipped with an NPN-type fiber optic amplifier, comprises a matrix sensor group designed to monitor seed flow during leakage from the planting machine. It utilizes infrared light-emitting diodes for emission and infrared photosensitive diodes for reception. The sensor has a minimum monitoring diameter of 0.4 mm and a maximum detection range of 70 mm × 110 cm. It effectively monitors seed flow after each opposing cutter operation, with a response time of less than 50 μs, and can differentiate pulse signals generated by various seeds.

To accurately measure the rotation angle intervals of the planter relative to the cutter shaft, an Omron E6B2-CWZ5B incremental rotary encoder (with a resolution of 1000 pulses per revolution) is employed. Fault alarm is a critical function of the seeding quality monitoring system. Given that cassava cultivation involves a real-time cut-and-mulch planting mode, it is essential to halt the planting operation promptly for inspection and troubleshooting in the event of seed shortages or sticky cutting sections. This study implements an alarm function to address two major faults: seed leakage and cutting section sticking. When a fault is detected by the system, the microcontroller drives the red and blue LED light-emitting modules to flash at varying intervals as part of the acoustic and optical alarms. Simultaneously, the system generates pulse-width modulation (PWM) signals with varying duty cycles to control the speakers, thereby providing acoustic and optical alarms. The components are electrically interconnected via power lines, signal lines, and communication lines to facilitate information acquisition and interaction. Specifically, the power line connects the tractor’s onboard 12V DC power supply to each piece of equipment, while the communication line utilizes an RS485 serial communication network.

#### 2.2.3 Seed metering algorithm

The seeding quality monitoring system is predicated on a matrix fiber optic sensor array and an incremental rotary encoder, which monitors the change in signal levels as seeds pass through adjacent cutters. Under normal operating conditions, the matrix fiber optic sensor array outputs a low-level signal to the microcontroller; when the cassava seed passes, the sensor array triggers an interrupt and outputs a high-level signal. After the seed passes, the system outputs a low-level signal again. The microcontroller collects the pulse width via the timer and subsequently calculates the duration of the cassava seed’s passage. Based on the duration of the passage time, the microcontroller determines whether there is leakage during seeding or instances of adhesion in the cut sections, and it controls the alarm unit to produce appropriate auditory and visual signals. The maximum forward speed of the cassava planting machine designed by the research team is 1.39 m/s (5 km/h). The minimum spacing between individual seeds is 0.06 m, and the minimum time interval between the drop of two adjacent seeds is


(1) $$T_{min}=\frac{S_{min}}{V_{max}}$$

Where T_min_ is the minimum seeding interval, s; S_min_ is the minimum planting spacing of single grain, m; V_max_ is the maximum forward speed of the planter, m/s.

Calculations indicate that the minimum seeding interval Tmin=0.043s=43ms, which is significantly larger than the duration of one cycle of the microcontroller control program (1–1.5 ms). Therefore, the developed program satisfies the requirements for single-grain seeding intervals.


(2) $$\theta =\frac{M}{R}\times 360^{\circ }$$

The cutter rotary axis of the cassava planter consists of three pairs of cutters; therefore, the rotation angle of each neighboring pair of cutters is set to 120°. Consequently, the monitoring window for the number of seeds sown is defined by this rotation angle, with each monitoring window corresponding to one seed sown. The real-time rotation angle of the planter’s cutter shaft is measured using a rotary encoder. During operation, the number of pulses generated by the rotary encoder is recorded in real time through a high-speed counter, enabling the determination of the rotation angle of the cutter shaft based on the encoder’s resolution.

where θ is the theoretical angle of rotation of the rotary axis of the grower cutter, °; M is the number of pulses of the encoder, and R is the resolution of the encoder, P/R.

The duration for which a cassava seed blocks light in the sensor monitoring area is related to the height from which the seed drops; this duration is closely associated with sowing quality. The cassava seeds complete the seeding process when they pass through a pair of cutters, with these cutters serving as the initial reference position. The time for the seed’s head and tail to reach the sensor monitoring region is calculated as follows


(3) $$h=\frac{1}{2}gt_{1}^{\;2}$$

(4) $$h+l=\frac{1}{2}gt_{2}^{\;2}$$

Where h is the height at which the first end of the cassava seed starts to fall to the monitoring area when it leaves the opposite cutter, m; l is the length of the cassava seed cut off, m; g is the acceleration of gravity, m/s^2^; t_1_ is the time used by the first end of the cassava seed to arrive at the monitoring area, s; and t_2_ is the time used by the last end of the cassava seed to arrive at the monitoring area, s.

The time t_s_ at which the cassava seed as a whole passes through the monitoring area is


(5) $$t_{s}=t_{2}-t_{1}=\frac{\sqrt{2\left(h+l\right)}-\sqrt{2h}}{\sqrt{g}}$$

Through experimental testing involving the use of a cutting device with three pairs of cutters, the minimum length of the cassava mechanized planting section is determined to be lmin=0.13m and the standard length is l=0.17m. When the seed section departs from the opposite cutter, its leading edge begins to drop to the monitoring area at a height of h=0.05m. Using the gravitational acceleration g=9.8m/s2 and substituting lmin=0.13m into Equation 5, calculated that the minimum seed completely blocked the beam time required for 0.09s. The selected fiber optic sensor has a response time of less than 200 μs, which meets the monitoring requirements for cassava seeding quality. Similarly, substituting l=0.17 m into Equation 5 yields that the time required for the standard-sized seed to completely block the beam is ts=0.11 s.

Combined with the theoretical spacing to determine the planting machine work sowing state (Abdolahzare and Abdanan Mehdizadeh, 2018), according to GB/T6973-2005 “single grain (precision) planter test methods” can be seen, when the actual spacing is less than or equal to 0.5 times the theoretical spacing for resowing; the actual spacing is greater than 1.5 times the theoretical spacing for missing, between the two for qualified, that is, the

Resowing


(6) $$d\leq 0.5d_{0}$$

Missing


(7) $$d>1.5d_{0}$$

Qualifying


(8) $$0.5d_{0}<d\leq 1.5d_{0}$$

Where d_0_ is the theoretical plant spacing, m; d is the actual plant spacing, m.

**Figure 5:**
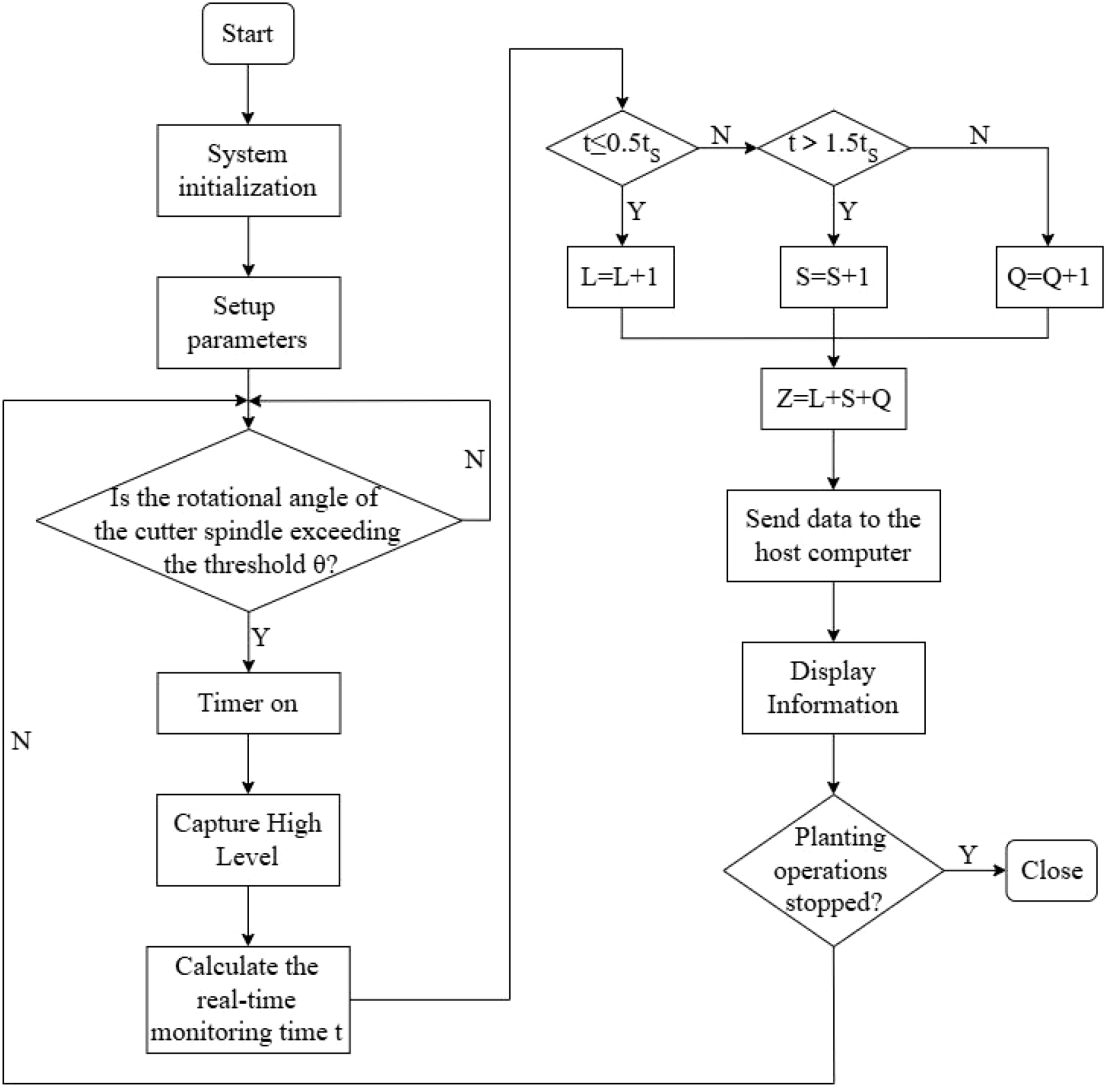
Flowchart for calculating seeding parameters. (L is the amount of missed seeding; S is the amount of cut section sticking; Q is the amount of qualified seeding; Z is the total amount of seeding; θ is the rotation angle threshold of the cutter rotary axis, 120°; t is the actual time of the seed section passing through the monitoring area, s; t_s_ is the theoretical time of the standard seed section passing through the monitoring area, s.).

In practice, within the rotation angle monitoring window of two adjacent cutters, 0.5 times the time interval t_s_ is set as the threshold for missed seeding detection, while 1.5 times t_s_ is used as the threshold for cut section sticking detection. If t ≤ 0.5ts,the planter is judged to have missed sowing; if t>1.5ts,it is judged to have cut section sticking; if 0.5ts<t ≤ 1.5ts,the planter is considered to be performing acceptable sowing. The parameter calculation flow is illustrated in **Figure 5**.

## 3 Testing of seeding quality monitoring system

### 3.1 Monitoring of sowing parameters and comparative trials

**Figure 6:**
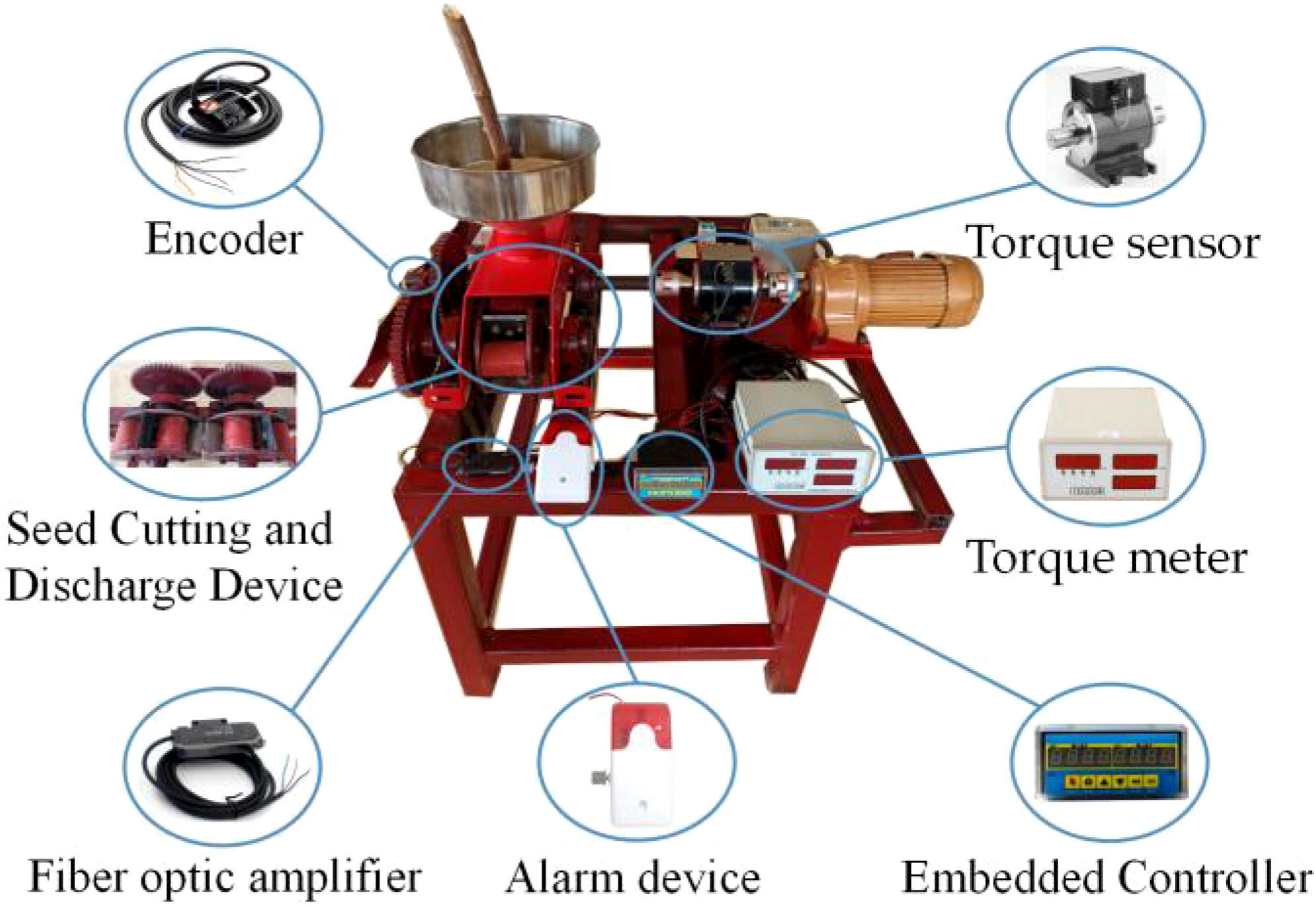
Seeding quality monitoring test device.

To verify the stability and accuracy of the system, a calibration test was conducted at various working speeds of the rotary axis of the opposing cutter. South Plant 199 cassava seed stems were used as test samples. Specifically, one hundred cassava seed stems were randomly selected, with an average moisture content of 37.65%. The length of these seed stems ranged from 1.4 to 1.7 meters, and their diameters ranged from 16 to 28 millimeters. The test equipment included a real-time seed cutting device, a monitoring system, a computer, dynamic torque transducers, a drive motor, and a motor frequency converter, as shown in **Figure 6**. The test was performed at the Key Laboratory of Agricultural Equipment for Tropical Crops, Ministry of Agriculture and Rural Affairs, Agricultural Machinery Research Institute, Chinese Academy of Tropical Agricultural Sciences.

In the test, according to GB/T6973-2005 “Test Methods for Single Grain (Precision) Seeders,” the rotational speed of the rotor shaft of the opposing cutter was chosen as the test factor (Xie et al., 2021c), with speeds of 10 r/min, 15 r/min, 20 r/min, 25 r/min, and 30 r/min. This speed gradient was representative of the actual working conditions for the cassava joint planting machine. The manual method was used to determine the actual number of cassava seed segments cut by the real-time seed cutting device and compare this with the values recorded by the monitoring system. Absolute and relative errors in sowing volume were calculated to assess the stability and accuracy of the monitoring system. Each test involved measuring 200 cassava seed segments, and the procedure was repeated three times.

### 3.2 Fault alarm response reliability test

The reliability of the fault alarm response was assessed by confirming that the monitoring system could record and generate alarms accurately and in a timely manner when faults were encountered. The test simulated two of the most critical and frequently occurring faults: a lack of cassava seed stems in the seed-cutting device and sticking due to incomplete cuts in the opposite sections of the seed-cutting device. The operation of the seeding quality monitoring system was managed through the start-stop button on the controller. During the seeding process, when cassava seed stems were not manually inserted while the seed-cutting device operated normally, it indicated that no seeds were dropped, simulating a seeding failure of the planter. Additionally, cassava seed stems were manually added to the hopper, the seed-cutting device operated normally, and the relative position and sharpness of the opposite cutter were adjusted to simulate incomplete cuts, representing the sticking fault. The two fault simulation tests were repeated 100 times each. During the tests, the accuracy of the fault alarm response was evaluated by observing if the acoustic and visual alarms activated when faults occurred, and whether the missed seeding and cut-section sticking were accurately displayed on the digital module. Simultaneously, a stopwatch was used to record the time elapsed from the occurrence of the fault to the monitoring system’s response.

### 3.3 A trial of real-time online monitoring of seeding parameters for planters in field operations

**Figure 7:**
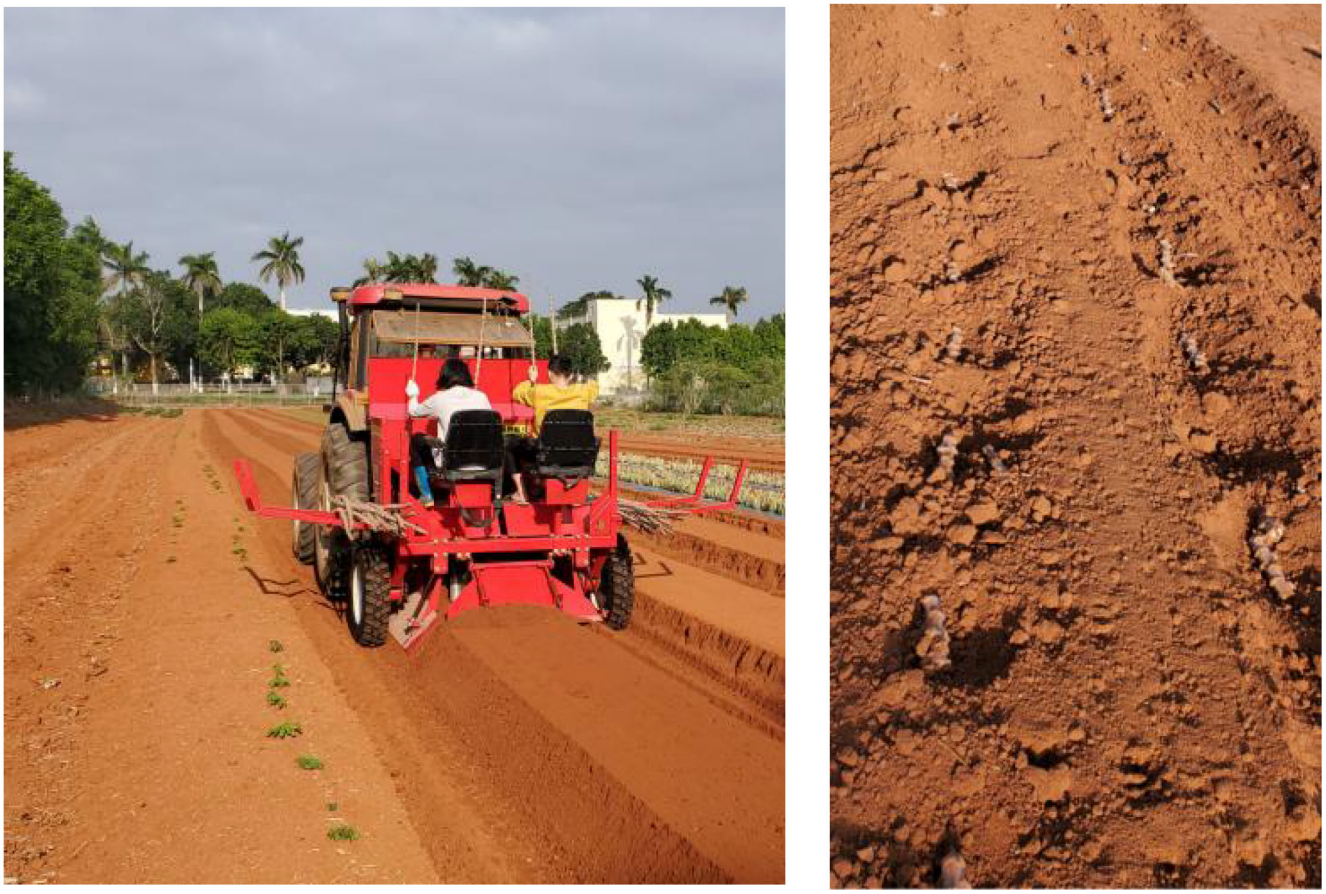
Field test.

The seeding quality monitoring test described above was conducted at a fixed rotational speed. During field operations, the rotational speed of the opposing cutter rotor shaft varies in real-time with the ground wheel drive of the combine planter, which results in more complex field operating conditions. To assess the effectiveness of the seeding quality monitoring system during actual field operations, a performance test of the monitoring system was carried out in Zhanjiang City, Guangdong Province, as illustrated in **Figure 7**. The test equipment and materials included cassava seed stems of South Plant No. 199, a starter cassava combine planter, a Dongfanghong 904 tractor, a monitoring system, a tape measure, a shovel, and other related items.

The influence of lighting conditions on the seeding quality monitoring system was examined prior to field seeding operations. The combine planter was elevated off the ground, and the seed cutting device was operated at 18 r/min under two conditions: no seed pole feeding and normal seed pole feeding. The monitoring system was activated, and tests were conducted under sunlight, artificial lighting, and shaded natural light conditions, respectively. The test results indicated that, under all three conditions, the terminal of the monitoring system recorded a value of zero when there was no seed rod feeding. Furthermore, the average relative error in seeding volume monitoring was 0.73% when the seed rods were fed normally. This suggests that natural light conditions in the field have minimal impact on the operation of the monitoring system.

During the test, in accordance with GB/T6973-2005 Test Methods for Single Grain (Precision) Planters, 20 meters were allocated at both the front and rear ends of the test field for speed adjustment of the planter and tractor. Additionally, 150 meters were designated as the data collection area for the planter’s stable operation. Based on the actual operating conditions of the combine planter, the tractor’s forward speed was set at three target speeds: 2 km/h, 3 km/h, and 4 km/h. Following sowing, the soil layer was manually removed to determine the total number of seeds sown by the planter. The effectiveness of the monitoring system was evaluated by comparing the system’s count with manually obtained results. Each test group was repeated three times, and the average value was calculated.

## 4 Results and discussion

The sowing quality monitoring system was fully and accurately verified through indoor bench tests and field tests, and the results of each test were analyzed as follows:

### 4.1 Monitoring of sowing parameters and analysis of results of comparative trials

**Figure 8:**
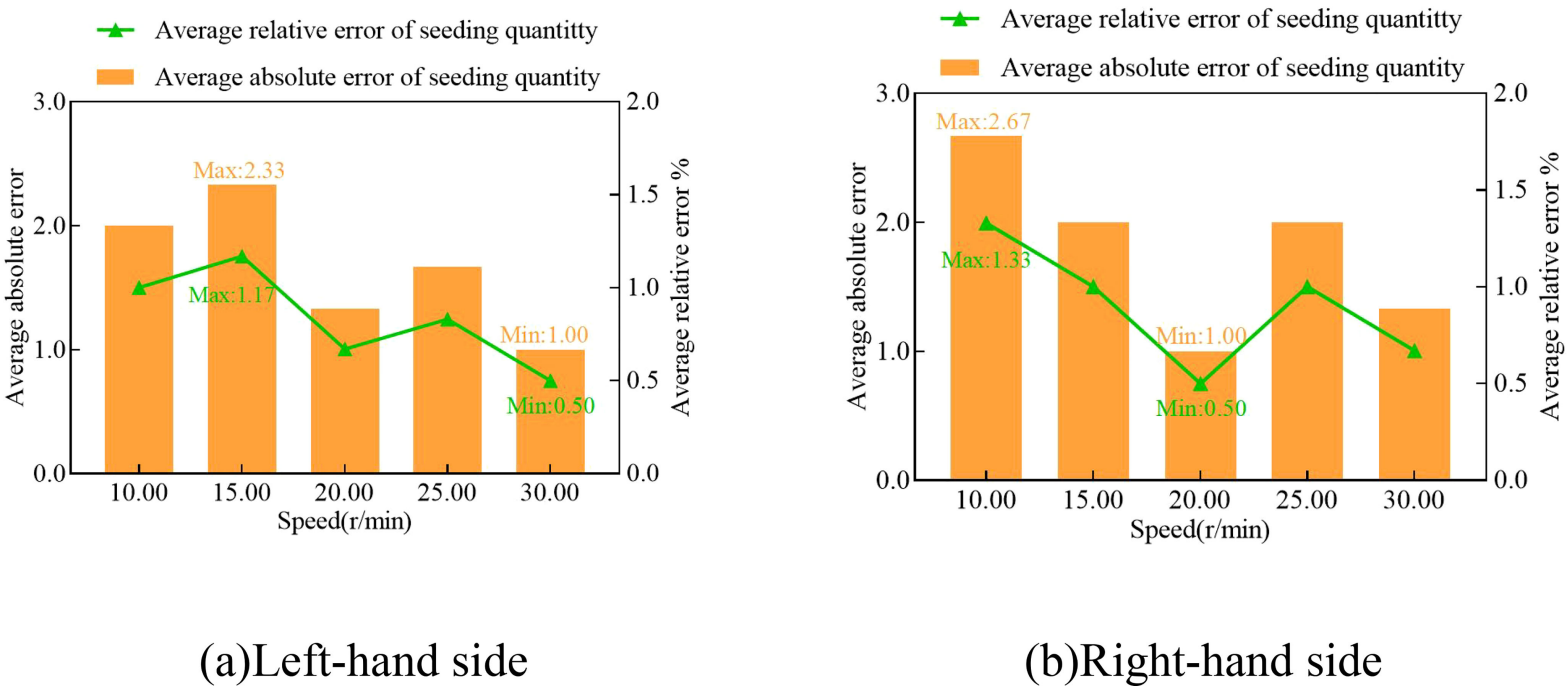
Seeding parameter monitoring comparison test results. **(A)** Left-hand side test results. **(B)** Right-hand side test results.

The results of monitoring the sowing volume of the cassava seed pole cutting device at various rotational speeds of the opposing cutter rotary axis are presented in **Table 1** and **Figure 8**. Throughout the test, the system did not experience any white crash screens or data transmission failures. Based on **Table 1** and **Figure 8**, the average absolute error between the monitored sowing volumes of the left and right rows and the actual values is ≤ 2.67, while the average relative error is ≤ 1.33%. These results demonstrate that the system exhibits high monitoring accuracy and satisfies the practical requirements.

**Table 1:** Results of seeding count trials.

Rotational speed of a rotating shaft(r/min)	Number	Left-hand side			Right-hand side		
		Actual number	Number of monitors	Relative error(%)	Actual number	Number of monitors	Relative error(%)
10	1	200	199	0.50	200	199	0.50
	2	200	197	1.50	200	197	1.50
	3	200	198	1.00	200	196	2.00
15	1	200	197	1.50	200	199	0.50
	2	200	198	1.00	200	197	1.50
	3	200	198	1.00	200	198	1.00
20	1	200	198	1.00	200	199	0.50
	2	200	199	0.50	200	200	0
	3	200	199	0.50	200	198	1.00
25	1	200	199	0.50	200	198	1.00
	2	200	198	1.00	200	199	0.50
	3	200	198	1.00	200	197	1.50
30	1	200	200	0	200	197	1.50
	2	200	199	0.50	200	200	0
	3	200	198	1.00	200	199	0.50

In order to further investigate the influence of different rotational speeds of the opposing cutter rotor shaft on the monitoring accuracy of the system, the relative error of the sowing volume of the left and right rows was analyzed as a function of the rotational speed of the cutter rotor shaft, using IBM SPSS Statistics software for analysis and Duncan’s Multiple Comparison Analysis for data processing. The results are presented in **Table 2**. The results indicated that the P-value for seeding volume in the left row across five rotational speeds was 0.306, and the P-value for the right row was 0.538. Both P-values were significantly greater than 0.05, indicating that the differences were not statistically significant. This further indicates that the monitoring accuracy of the system remains relatively stable within the tested range of 10 to 30 r/min for the cutter spindle’s rotational speed, and the impact of rotational speed on the monitoring accuracy is minimal, thus meeting the requirements for normal operation.

**Table 2:** Analysis of variance of seeding quantity under speed processing.

Monitoring channel	Cognitive	Sum of squares	df	Mean square	F	P
Left-hand side	Between groups	3.333	4	0.833	1.389	0.306
	Within groups	6.000	10	0.600		
	Total	9.333	14			
Right-hand side	Between groups	5.067	4	1.267	0.826	0.538
	Within groups	15.333	10	1.533		
	Total	20.400	14			

In summary, the seeding quality monitoring system developed by this institute demonstrates an accuracy comparable to manual statistics, with a minimal relative error. Moreover, the monitoring accuracy remains stable under varying rotational speeds, with minimal influence from the cutter spindle’s rotational speed, thus meeting practical requirements.

### 4.2 Analysis of fault alarm response reliability test results

**Figure 9:**
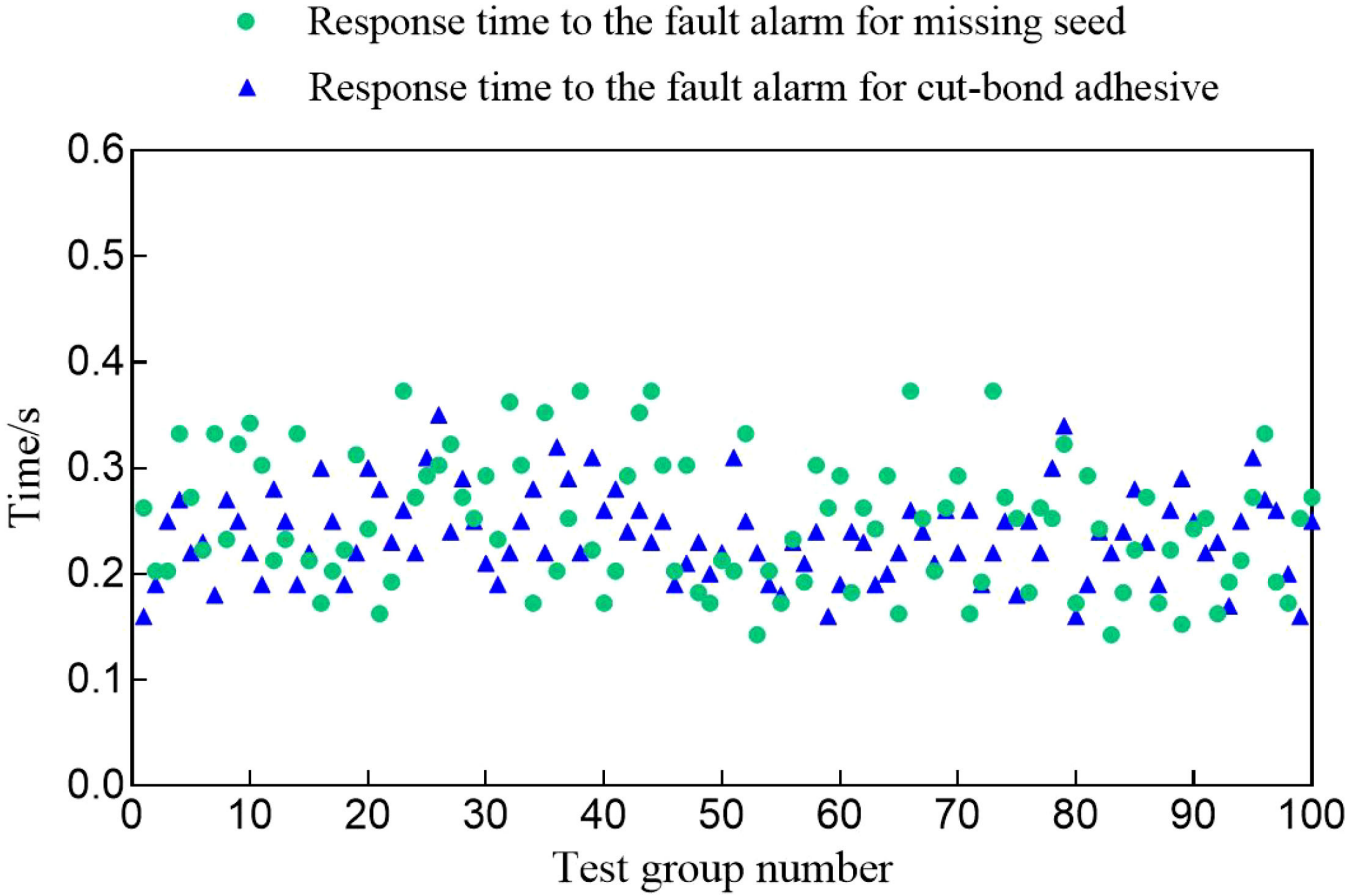
Scatter plot of the response time of monitoring system fault alarm.

The fault simulation response test encompassed both missing seed segments and sticky seed segments, with the time interval from the initiation of the fault to the system alarm being recorded. A missing seed segment occurs when the manual seed release is delayed, causing the combine harvester to continue operating without any change in the system level within the monitoring window. Seed segment sticking involves a change in the relative position or sharpness of the seed cutting device’s blade, leading to incomplete cutting and resulting in seed segments remaining in the monitoring system longer than the typical drop time. Test results indicate a 100% success rate for fault detection, with no instances of missed fault alarms, demonstrating the reliability of the system’s audible and visual alarm functions. The scatter plot of fault alarm response times for the monitoring system is presented in **Figure 9**. Assuming that the manual timing response is negligible, the figure demonstrates that the response times for both faults are relatively stable, with an average value of less than 0.4 seconds. Therefore, the fault alarm response time of the sowing quality monitoring system is minimal and meets the requirements for practical operation. When a seeding operation fault occurs, the monitoring system provides timely and accurate alarms, alerting the operator to stop and inspect the equipment to prevent further economic losses.

In summary, the seeding quality monitoring system designed by this institute has a 100% success rate in response to faults when there are faults of missing seed segments and sticky seed segments, and the response time of both faults is relatively stable, with an average value of less than 0.4s, so that timely and accurate alarms can be issued.

### 4.3 Analysis of experimental results for real-time online monitoring of planter seeding parameters in field operations

The results of real-time monitoring of sowing parameters during the field operation of the cassava combine planter are presented in **Table 3**. The accuracy and reliability of the monitoring system under actual field conditions were validated through tests involving human-induced missed sowing and adjustments to the relative position and sharpness of the cutter. The table shows that the relative error interval for seeding volume monitoring ranged from 0.48% to 1.46%, with an average relative error of 0.91%, within the operating speed range of 2 to 4 km/h for agricultural implements. The fault alarm response success rate was 100%. The field tests demonstrate that the monitoring system remained operational over extended periods, with measurement results consistently stable. The system effectively monitored agricultural equipment across varying operating speeds and met the practical work requirements.

**Table 3:** Field performance experiment results of monitoring system.

Forward speed(km/h)	Rotational speed of a rotating shaft(r/min)	Number of seeds to be sown	Number of qualifications	Number of monitors	Relative error (%)	Number of faults (missed seeding and cutted section sticking)	Fault alarm rate (%)
2	11.89	215	202	200	0.99	13	100
		218	206	203	1.46	12	100
		220	209	207	0.96	11	100
3	17.84	221	207	205	0.97	14	100
		216	206	205	0.49	10	100
		220	208	207	0.48	12	100
4	23.78	224	214	211	1.42	10	100
		217	205	204	0.49	12	100
		225	213	211	0.94	12	100

## 5 Conclusions

This paper addresses a seeding quality monitoring system specifically designed for real-time cut-seed planters used for cassava. This research area has predominantly focused on monitoring the sowing of small and medium-sized seeds, and to date, no model or system for real-time cut-seed segment seeding quality monitoring has been identified. The starter cassava combine planter developed by the research team rarely suffers from ground wheel slippage in field operations. Therefore, the potential effects of ground wheel slippage have not been incorporated into the consideration of the system’s monitoring performance in the current research design. Accordingly, the system was able to accurately monitor the seeding parameters of the combine planter, including the total amount of seed sown, amount of seed passed, amount of seed missed, and amount of cut-section sticking. In the event of a planter failure, the system promptly activates both sound and light alarms to prevent losses due to missed seeding and cut-section sticking. The fiber optic sensor exhibits strong dust penetration capabilities, making the monitoring system less susceptible to the complex operating environment in the field. However, since the entire system relies on light interactions, it appears infeasible to fully eliminate the impact of dust. In the complex field environment, the sensors are prone to dust accumulation during prolonged monitoring, which may reduce monitoring accuracy. The primary conclusions are as follows:

1. Based on an analysis of the working process of the seed-cutting device in the combine planter, a seeding quality monitoring system for cassava combine planters was designed. This system employs opposed fiber-optic sensors and rotary encoders as the primary monitoring elements, details the structure and working principle of the system, and establishes monitoring algorithms for each seeding state based on three potential operational scenarios in real-time seed-cutting operations.
2. According to the indoor test results, when the simulated rotational speed of the opposing cutter ranges from 10 to 30 rpm, the average absolute error between the monitored sowing volumes of the left and right rows and the actual values is 2.67, with an average relative error of 1.33%. Duncan’s multiple comparison analysis indicates that the differences between the system’s monitored values and manually measured values are not significant. The system’s fault alarm response function is both accurate and timely, achieving a 100% alarm success rate and an average response time of less than 0.4 seconds. The indoor bench tests demonstrate that the monitoring system maintains high and relatively stable accuracy, detects faults promptly and accurately, and exhibits minimal sensitivity to variations in sowing speed.
3. According to the results of the field test, natural light conditions do not affect the performance of the monitoring system, which operates effectively in the complex field environment. At operating speeds of 2 to 4 km/h, the relative error range for seeding amount monitoring is between 0.48% and 1.46%, with an average relative error of 0.91%. The alarm success rate is 100%. The monitoring system’s error is within a reasonable range, making it suitable for monitoring actual seeding operations.

Future research will explore the use of dust-proof components or clean fiber optic sensors, such as the installation of dust-proof glass or the integration of a self-detection system to optimize sensor dust levels and mitigate the negative impact of dust on monitoring accuracy. Additionally, automation and multi-source information fusion technologies will be integrated to develop an intelligent seed replenishment system, enabling precise seeding even in cases of missed seeding, thereby better aligning the system with real-world field production conditions.

## Data Availability

The datasets presented in this study can be found in online repositories. The names of the repository/repositories and accession number(s) can be found in the article/supplementary material.
